# Predictors of attrition among adults in a rural HIV clinic in southern Mozambique: 18-year retrospective study

**DOI:** 10.1038/s41598-021-97466-2

**Published:** 2021-09-09

**Authors:** Edy Nacarapa, M. Elisa Verdu, Joana Nacarapa, Artur Macuacua, Bartolomeu Chongo, Dulce Osorio, Isabelle Munyangaju, Didier Mugabe, Roger Paredes, Ana Chamarro, Boris Revollo, Silvio S. Alexandre, Mulassua Simango, Diego Torrus, Jose-Manuel Ramos-Rincon

**Affiliations:** 1 Carmelo Hospital of Chókwè – The Daughters of Charity, Saint Vicente of Paul, TB/HIV Division, Avenida Trabalho, Chokwé, Gaza Province Mozambique; 2 Tinpswalo Association, Vincentian Association to Fight AIDS and TB, Research Unit, Chókwè, Gaza Province, Mozambique; 3 Macia Health Centre, Macia, Gaza Mozambique; 4 National Institute of Health, Maputo, Mozambique; 5 IrsiCaixa – Institute of AIDS Research, Barcelona, Spain; 6 FLS Foundation – Fight AIDS Foundation, Barcelona, Spain; 7 Gaza Health Provincial Direction, Xai-Xai, Mozambique; 8 Department of Internal Medicine, University General Hospital of Alicante and Miguel Hernandez University, Elche, Spain

**Keywords:** Medical research, Epidemiology

## Abstract

HIV remains a major cause of morbidity and mortality for people living in many low-income countries. With an HIV prevalence of 12.4% among people aged over 15 years, Mozambique was ranked in 2019 as one of eight countries with the highest HIV rates in the world. We analyzed routinely collected data from electronical medical records in HIV-infected patients aged 15 years or older and enrolled at Carmelo Hospital of Chokwe in Chokwe from 2002 to 2019. Attrition was defined as individuals who were either reported dead or lost to follow-up (LTFU) (≥ 90 days since the last clinic visit with missed medical pick-up after 3 days of failed calls). Kaplan–Meier survival curves and Cox regression analyses were used to model the incidence and predictors of time to attrition. From January 2002 to December 2019, 16,321 patients were enrolled on antiretroviral therapy (ART): 59.2% were women, and 37.9% were aged 25–34 years old. At the time of the analysis, 7279 (44.6%) were active and on ART. Overall, the 16,321 adults on ART contributed a total of 72,987 person-years of observation. The overall attrition rate was 9.46 per 100 person-years. Cox regression showed a higher risk of attrition in those following an inpatient regimen (hazard ratio [HR] 3.18, 95% confidence interval [CI] 2.89–3.50; *p* < 0.001), having CD4 counts under 50 cells/µL (HR 1.91, 95% CI 1.63–2.24, *p* < 0.001), receiving anti-TB treatment within 90 days of ART initiation (HR 6.53, 95% CI 5.72–7.45; *p* < 0.001), classified as WHO clinical stage III (HR 3.75, 95% CI 3.21–4.37; *p* < 0.001), and having Kaposi’s sarcoma (HR 1.99, 95% CI 1.65–2.39, *p* < 0.001). Kaplan–Meier analysis showed that patients with CD4 counts of less than 50 cells/µL on ART initiation had a 40% lower chance of survival at 18 years. Low CD4 cell counts, ART initiation as an inpatient, WHO clinical stage III, and anti-tuberculosis treatment within 90 days of ART initiation were strongly associated with attrition. Strengthening HIV testing and ART treatment, improving the diagnosis of tuberculosis before ART initiation, and guaranteed psychosocial support systems are the best tools to reduce patient attrition after starting ART.

## Introduction

Mozambique is a low-income country in southern Africa with among the highest HIV prevalence in the world, estimated at 12.6% among adults (15–49 years old)^1,2^. In 2020, there were an estimated 2.1 million people living with HIV (PLWHIV) in the country^3^, up from the 1.5 million who had been diagnosed in 2010^4^. Likewise, the number of people on antiretroviral therapy (ART) nearly quadrupled from 2012 to 2017 (309,000–1.2 million)^5,6^. Therefore, significant progress in the fight against HIV/AIDS has been made with the support of programs and bodies such as the Global Fund, the President’s Emergency Plan for AIDS Relief (PEPFAR) and the Mozambican Ministry of Health. However, though Mozambique achieved treatment coverage of more than 55% by the end of 2018^3^, this is far from the 95% recommended by UNAIDS^7^.

The attrition rate (resulting from mortality or loss to follow-up [LTFU]) remains one of the key challenges to the success of the Mozambican national ART program. Data from 94 health facilities from all the Mozambican provinces revealed that the overall mortality rate was 3.4 deaths per 100 person-years, and 12.9 deaths per 100 person-years in the first 90 days. Attrition rates were 19.8 and 57.2 losses per 100 person-years, respectively^8^. Data from multi-country ART programs in 350 health facilities in four other Eastern African countries (Ethiopia, Kenya, Mozambique, and Tanzania) from 2005 to 2014 reported attrition rates of 26.2% at 12 months, 34.0% at 24 months, and 40.1% at 36 months after starting ART^9^. Other data from a large multi-country program in 198 HIV clinics in Kenya (69 clinics), Mozambique (33 clinics), Rwanda (44 clinics) and Tanzania (52 clinics) revealed that the sex-related differences in the relationship between age, mortality and LTFU applied to both younger patients on ART as well as to older patients aged 50–59 years, and that women on ART appear to have disproportionate risk of death as they age compared to men^10^.

Known predictors of attrition include low initial CD4 T-cell lymphocyte counts, low baseline body mass index (BMI), history of hospitalization, advanced WHO clinical staging, older age at HIV diagnosis, and being a man^11–13^. Other studies from the region show anemia and decreased red blood cell counts and platelets as predictors of death during the first year of ART treatment^14^.

To continuously evaluate the success of ART programs in Southern Africa, data on attrition, one of the key WHO reportable indicators, should be periodically reported. Until now, there has been a paucity of data to describe attrition from ART care in Chokwe District, Mozambique. This study aimed to describe the incidence and predictors of attrition (death and loss to follow-up) in adults initiating ART in a rural HIV clinic of Carmelo Hospital Chókwè (CHC), Chokwe District, Mozambique.

## Methods

### Study design and population

**Figure 1:**
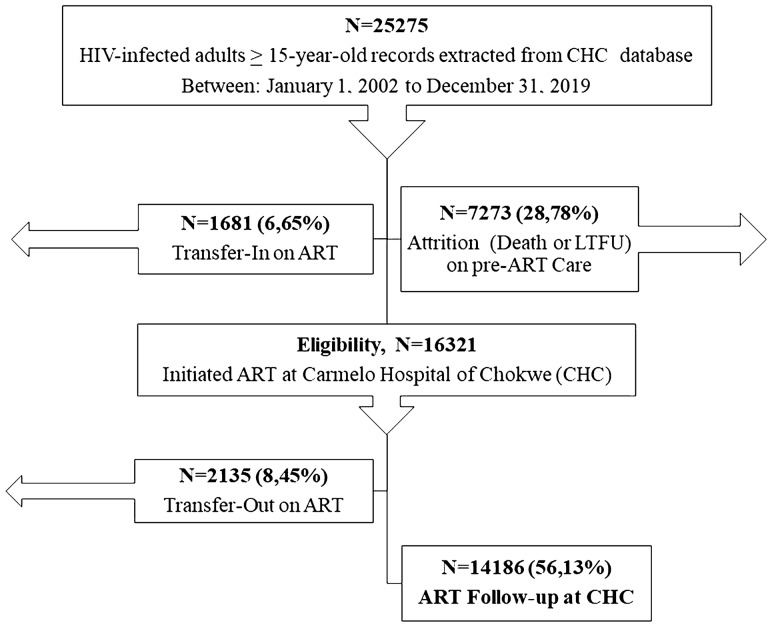
Patient flowchart.

This retrospective cohort study included data for all HIV-infected individuals aged 15 years or older who initiated ART at the CHC between January 2002 and December 2019. Individuals who were referred to the CHC after initiating ART in a center other than CHC were excluded from the analysis (n = 1681) (Fig. 1).

### Setting

CHC is a state-owned reference hospital in the district of Chókwè, a large rural district in Gaza Province in southern Mozambique, administered by Catholic missionaries (the Daughters of Charity, Saint Vincent de Paul) since 1993. Chókwè is predominantly rural, with a surrounding catchment population of approximately 200,000 inhabitants. Agriculture is the sole source of income for roughly 80% of the population, and approximately 40% of the population migrates seasonally to South Africa, seeking work in the mines. According to the last national HIV prevalence study in 2015, Gaza Province had the highest provincial HIV prevalence in the country at 24.4%^2^.

### Definitions

We classified the ART outcomes as retention or attrition. Retention (encompassing adherence) was defined as documented active and alive HIV-infected adults who were still on ART (assessed at intervals longer than 6 months post-initiation). Attrition was defined as death and LTFU*.*

Our reported mortality rate represents the number of documented deaths during ART per 100 person-years of observed therapy. The attrition rate represents the number of patients lost through attrition (death, LTFU) per 100 person-years. Patients were considered LTFU if they did not show up for their consultation in the 90 days preceding data abstraction, with missed treatment pick-up after 3 days of failed calls, unless the medical record stated that the patient had died, stopped ART, or was transferred out. The date of LTFU was recorded as the date of the last recorded visit, or 1 day after ART initiation if the patients never returned after ART initiation. To enable comparison with published literature, we report 1-year attrition^15,16^. Patients transferred to other facilities during the 18 treatment years were excluded from the 18-year attrition proportion. Transfers were censored in time-to-event analyses at the date of transfer. To facilitate survival analysis, we assumed that individuals who initiated ART but never returned over the 18 years of follow-up contributed 1 day of follow-up. Individuals remaining in care were censored at the end of the 18-year study period. Individuals who were either reported dead, LTFU or had transferred to other health care facilities were censored at their last clinic visit date. To allow comparison with other ART programs in low-resource settings, the first CD4 count was used to estimate the treatment outcome^17,18^.

CD4 counts were determined using the FlowCount PLG CD4 assay and analyzed with Beckman Coulter Epics XL-MCL Flow Cytometers (both Beckman Coulter, Inc., Johannesburg, South Africa). Blood hemoglobin was determined with an automated hematology analyzer, Sysmex XT2000i (Sysmex Europe, Inc., Hamburg, Germany) or HemoCue photometer assay (HemoCue AB, Helsingborg, Sweden).

Similar to other cohort studies in resource-constrained settings^16^, adherence to ART was estimated by measuring timeliness of patient visits to scheduled medicine pick-up appointments at clinic-based pharmacies^19,20^ during the first 6 months of ART.

Categories used to classify exposure to anti-tuberculosis treatment (ATT) were: 1) completed ATT before ART initiation: 2) initiated ATT < 90 days after ART initiation; and 3) unexposed to ATT at ART initiation.

### Data collection

Data collection was performed using DREAM software (Diseases Relief through Excellence and Advanced Means), version 6.1.0.757, and electronic medical record (EMRs), designed according to criteria of excellence to manage the prevention and treatment centers of the DREAM Program of Sant'Egidio Community, located in 11 African countries, including Mozambique. Since 2002, the Catholic missionary organizations Daughters of Charity Saint Vincent de Paul (http://www.daughtersips.org/hivaids) and the Community of Sant'Egidio (http://www.dream.santegidio.org) have a collaboration agreement that allows the Daughters of Charity health facilities to use the DREAM software to manage their HIV-infected patients. Carmelo Hospital of Chokwe is a health facility administered by the Daughters of Charity and uses the DREAM Dataset as main the main EMR system. It works through Microsoft Access and JavaScript and is supervised by the hospital's local IT department. All offices work electronically and use the database for routine consultations, so all patient information is automatically stored on a common server. The ART database collects and stores long-term EMRs, sociodemographic data, clinical histories, laboratory tests (hematology, biochemistry), identification codes, scheduling of new appointments, and pharmaceutical stocks.

Recorded data from eligible ART participants for the study were collected and anonymized to remove identifying details: each patient was assigned an alphanumeric code (ID), and the anonymous data were included in the data sheet.

To convert data from Microsoft Access to SPSS, the Access data were first exported to a Microsoft Excel spreadsheet, and in turn the spreadsheet data were imported to SPSS for subsequent data analysis.

For each patient, we included the following data: gender, age (age band and median), point of entry (inpatient or outpatient), CD4 count at baseline, previous ART exposure, previous ATT (ATT prior to ART initiation, ATT within 90 days of ART initiation, and no exposure to ATT), WHO clinical stage of HIV disease, Kaposi’s sarcoma diagnosis, ART therapy, and final ART outcome.

### Data analysis

Statistical analysis was performed using IBM SPSS Statistics Software version 25 (IBM Corporation, International Business Machines Corp, Release 2017, https://www.ibm.com/legal/copytrade, USA). Patients’ baseline characteristics, as described above, were compared according to outcomes.

Time-to-event methods were used to investigate predictors of attrition. Time was measured from ART initiation to death or LTFU. Patients still alive at the date of data collection were considered active in ART. Patients who did not come to the clinic for at least 90 days after their last scheduled appointment were considered LTFU^18^. Attrition was calculated by summing the number of patients who experienced the event (deaths, LTFU) during a particular period divided by the total years of follow-up during this period. Multivariable analysis was performed by Cox regression models. The proportional hazard assumption was assessed with log survival curves based on Schoenfeld residuals. We used Kaplan–Meier survival estimates to calculate the cumulative incidence of attrition. The cumulative incidence of mortality and LTFU were calculated using competing risk analysis.

The Mozambican National Bioethics Committee for Health (*Comité Nacional de Bioética para a Saúde,* 25/CNBS/2019) approved this analysis. Analysis was performed on de-identified, aggregated patient level data, and no individual informed consent was obtained. The need for written informed consent was explicitly waived, and the research was performed in accordance with the Declaration of Helsinki.

### Ethics approval and consent to participate

The Mozambican National Bioethics Committee for Health (*Comité Nacional de Bioética para a Saúde,* 25/CNBS/2019), approved this analysis (25/CNBS/2019). Analysis was performed on de-identified, aggregated patient level data, and the need for written informed consent was explicitly waived.

### Consent for publication

We performed analysis on routine administrative data; consent for publication is not applicable.

## Results

### ART treatment outcomes

A total of 25,275 HIV-infected adults aged 15 years or older were enrolled in HIV care from 1 January 2002 to 31 December 2019. Of these, 16,321 (64.6%) initiated ART at CHC during the 18-year study period (Fig. 1).

**Figure 2:**
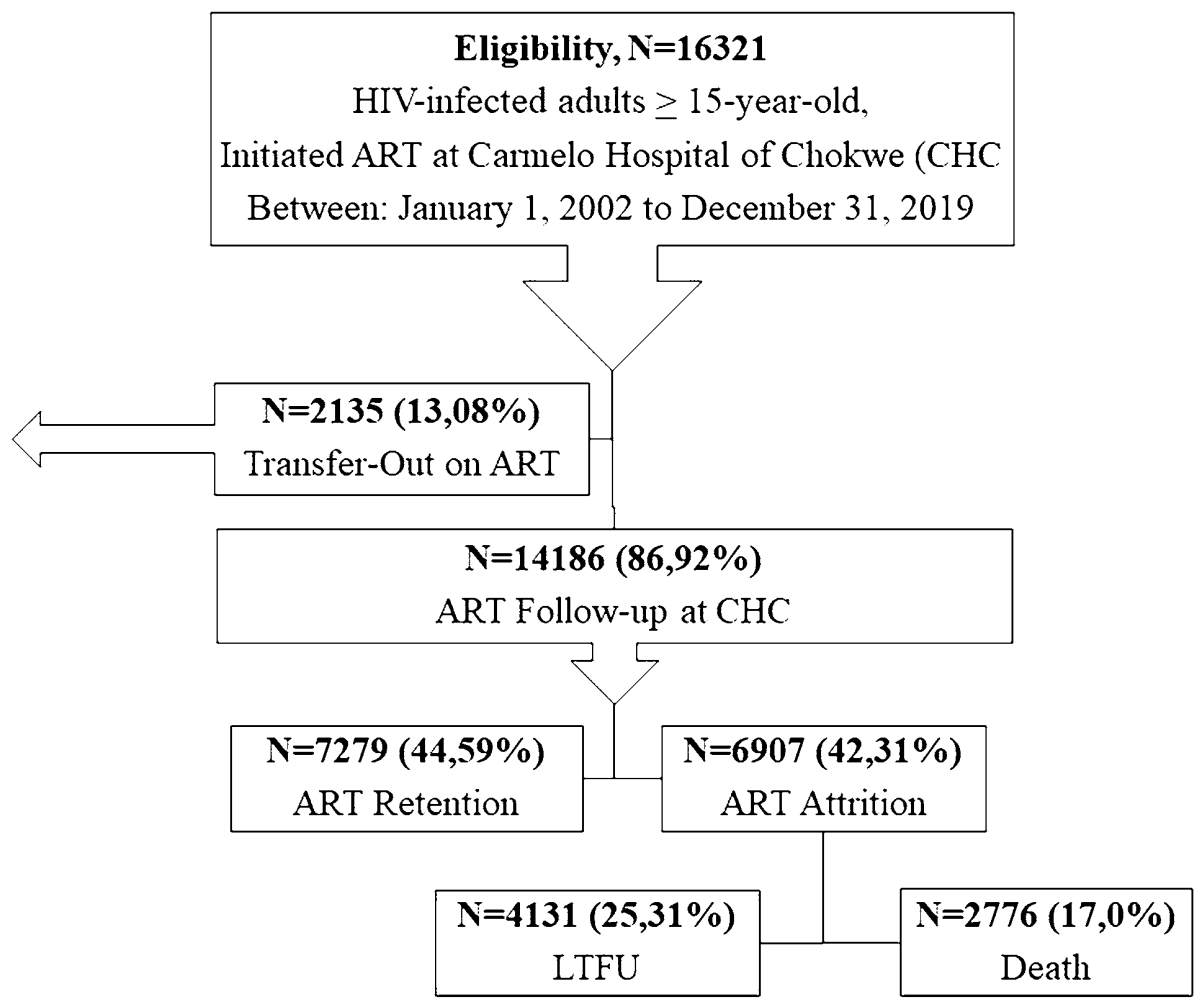
ART outcomes among PLWHIV at Carmelo Hospital of Chókwè (2002–2019), N = 16,321.

Of the total sample, 2135 (13.1%) were listed as transferred to another facility, while 7279 (44.6%) were active on ART during the study period (Fig. 2).

Median age at enrollment was 35 years (interquartile range [IQR] 15–93); 9657 (59.2%) were women, and 6190 (37.9%) were aged 25–34 years. Median follow-up was 9 years (IQR, 1.0–18.0 years). The vast majority (n = 15,454, 94.7%) were classified as ART-naive (Table 1).

**Table 1:** Characteristics of HIV patients enrolled at Carmelo Hospital of Chókwè of between January 1, 2002 and December 31, 2019 by Outcome Status.

N = 16,321	Total		Retention		Attrition			
					Death		LTFU	
	N	%	N	%	N	%	N	%
**Gender**								
Male	6664	40.80%	2361	32.40%	1448	52.2%	2023	49.0%
Female	9657	59.20%	4918	67.60%	1328	47.8%	2108	51.0%
**Age (years) , median (IQR)**	35	(15–93)	36	(15–84)	36	(15–93)	33	(15–82)
**Age band**								
15–24	1697	10.40%	628	8.60%	208	7.5%	586	14.2%
25–34	6190	37.90%	2618	36.00%	982	35.4%	1755	42.5%
35–44	4713	28.90%	2285	31.40%	786	28.3%	1085	26.3%
45–54	2490	15.30%	1216	16.70%	515	18.6%	447	10.8%
55 +	1231	7.50%	532	7.30%	285	10.3%	258	6.2%
**Point of entry**								
In-patient	2431	14.90%	315	4.30%	1578	56.8%	346	8.4%
Out-patient	13,890	85.10%	6964	95.70%	1198	43.2%	3785	91.6%
**CD4 + T-cell count (Cell/µL) , median (IQR)**	192	(0–2895)	256	(1–2895)	67	(0–1763)	197	(0–2802)
**CD4 + T-cell count (Cell/µL) Range**								
< 50	2713	16.6%	593	8.1%	1220	43.9%	597	14.5%
50–99	2858	17.5%	1173	16.1%	594	21.4%	676	16.4%
100–199	2804	17.2%	1135	15.6%	451	16.2%	821	19.9%
200–349	4665	28.6%	2299	31.6%	399	14.4%	1330	32.2%
350–499	1356	8.3%	737	10.1%	62	2.2%	414	10.0%
500 +	1925	11.8%	1342	18.4%	50	1.8%	293	7.1%
**Previous ART exposition**								
ART naive	15,454	94.70%	6944	95.40%	2627	94.6%	3843	93.0%
ART non-naive	867	5.30%	335	4.60%	149	5.4%	288	7.0%
**Previous Anti-TB Treatment (ATT)**								
Complete ATT before ART initiation	511	3.10%	224	3.10%	54	2.5%	152	3.7%
Initiated ATT < 90 days after ART initiation	4667	28.60%	1061	14.60%	2072	97.4%	1040	25.2%
Unexposed to ATT initiation	10,490	64.30%	5994	82.30%	1	0.0%	2937	71.1%
**WHO clinical staging of HIV disease**								
Clinical stage I	4501	27.60%	2787	38.30%	75	2.7%	1066	25.8%
Clinical stage II	3588	22.00%	1990	27.30%	119	4.3%	970	23.5%
Clinical stage III	2737	16.80%	1235	17.00%	165	5.9%	870	21.1%
Clinical stage IV	5495	33.70%	1267	17.40%	2417	87.1%	1225	29.7%
**Kaposi Sarcoma**								
Kaposi Sarcoma	299	1.80%	54	0.70%	133	4.8%	80	1.9%
No Kaposi Sarcoma	16,022	98.20%	7225	99.30%	2643	95.2%	4051	98.1%
**ART enrollment year**								
2002	8	0.00%	2	0.00%	4	0.1%	0	0.0%
2003	77	0.50%	42	0.60%	18	0.6%	6	0.1%
2004	63	0.40%	29	0.40%	21	0.8%	6	0.1%
2005	234	1.40%	94	1.30%	80	2.9%	25	0.6%
2006	785	4.80%	293	4.00%	239	8.6%	147	3.6%
2007	1232	7.50%	439	6.00%	348	12.5%	295	7.1%
2008	1282	7.90%	526	7.20%	284	10.2%	286	6.9%
2009	1273	7.80%	538	7.40%	277	10.0%	287	6.9%
2010	1469	9.00%	528	7.30%	285	10.3%	425	10.3%
2011	1205	7.40%	437	6.00%	237	8.5%	359	8.7%
2012	1398	8.60%	565	7.80%	207	7.5%	442	10.7%
2013	1030	6.30%	477	6.60%	101	3.6%	342	8.3%
2014	1732	10.60%	828	11.40%	194	7.0%	497	12.0%
2015	1415	8.70%	698	9.60%	152	5.5%	386	9.3%
2016	1164	7.10%	612	8.40%	140	5.0%	271	6.6%
2017	844	5.20%	431	5.90%	111	4.0%	194	4.7%
2018	674	4.10%	396	5.40%	65	2.3%	123	3.0%
2019	436	2.70%	344	4.70%	13	0.5%	40	1.0%
**ART therapy initiation**								
ABC + 3TC + EFV	56	0.30%	19	0.30%	25	0.9%	5	0.1%
ABC + 3TC + LPV/RTV	16	0.10%	4	0.10%	7	0.3%	4	0.1%
AZT + 3TC + ABC	203	1.20%	67	0.90%	48	1.7%	61	1.5%
AZT + 3TC + EFV	418	2.60%	190	2.60%	80	2.9%	114	2.8%
AZT + 3TC + LPV/RTV	52	0.30%	30	0.40%	8	0.3%	11	0.3%
AZT + 3TC + NVP	5511	33.80%	2750	37.80%	600	21.6%	1360	32.9%
D4T + 3TC + ABC	531	3.30%	70	1.00%	263	9.5%	143	3.5%
D4T + 3TC + EFV	666	4.10%	43	0.60%	344	12.4%	212	5.1%
D4T + 3TC + LPV/RTV	3	0.00%	1	0.00%	1	0.0%	1	0.0%
D4T + 3TC + NVP	1588	9.70%	138	1.90%	638	23.0%	531	12.9%
TDF + 3TC + DTG	68	0.40%	65	0.90%	0	0.0%	1	0.0%
TDF + 3TC + EFV	6767	41.50%	3631	49.90%	690	24.9%	1615	39.1%
TDF + 3TC + LPV/RTV	442	2.70%	271	3.70%	72	2.6%	73	1.8%

*ART* antiretroviral treatment, *ABC* abacavir, *3TC* lamivudine, *EFV* efavirenz, *LPV/RTV* lopinavir/ritonavir, *AZT* zidovudine, *D4T* stavudine; *NVP* nevirapine, *DGT* dolutegravir.

Outpatient consultations were the main source of entry (n = 13,890, 85.1%). At baseline, about a third of the patients (n = 5495, 33.7%) were classified as having WHO clinical stage IV disease. The median CD4 cell count was 192 cells/µL (IQR 0–2654), and nearly a third (n = 4665; 28.6%) of new enrollees had a CD4 count of 200–349 cells/µL. A similar proportion (n = 4667; 28.6%) were in TB treatment within the first 90 days of ART initiation, and 299 (1.8%) patients had Kaposi’s sarcoma (KS). A third (n = 5511; 33.8%) were under the AZT + 3TC + NVP regimen.

Of the 9042 (55.4%) patients with an unfavorable HIV treatment outcome, 2776 (17.0%) died, and 4131 (25.3%) were LTFU (Fig. 2). The proportion of deaths was significantly higher in adults aged 25–34 years (n = 982; 35.4%), those receiving ATT within 90 days of ART initiation (n = 2072; 97.4%), patients with CD4 counts of less than 50 cells/µL (n = 1220; 43.9%), men (n = 1448; 52.2%), those with WHO clinical stage IV of HIV disease (n = 2417; 87.1%), and people starting ART as inpatients (n = 1578; 56.8%);

### Patient characteristics by gender, age, and point of entry

Women comprised 59.2% (n = 9657) of the sample initiating ART at CHC; 40.2% (n = 3883) of the women were aged 25–34 years-old, 88.9% (n = 8589) presented to CHC to initiate outpatient the ART regimen; 32.6% (n = 3145) had CD4 counts of 200–349 cells/µL, and 32.8% (n = 3168) presented with WHO clinical stage I disease (*p* < 0.0001) (Table 2).

**Table 2:** Characteristics of HIV patients enrolled at Carmelo Hospital of Chókwè between January 1, 2002 and December 31, 2019 by Gender Status.

	Gender						*p* value
	Female		Male		Total		
	N	%	N	%	N	%	
**Age (years) , median (IQR)**	33	(15–93)	37	(15–91)	35	(15–93)	
**Age band**							< 0.0001
15–24	1362	14.1%	335	5.0%	1697	10.4%	
25–34	3883	40.2%	2307	34.6%	6190	37.9%	
35–44	2527	26.2%	2186	32.8%	4713	28.9%	
45–54	1341	13.9%	1149	17.2%	2490	15.3%	
55 +	544	5.6%	687	10.3%	1231	7.5%	
**Point of entry**							< 0.0001
In-patient	1068	11.1%	1363	20.5%	2431	14.9%	
Out-patient	8589	88.9%	5301	79.5%	13,890	85.1%	
**CD4 + T-cell count (Cell/µL) , median (IQR)**	228	(0–2895)	138	(0–2802)	192	(0–2895)	
**CD4 + T-cell count (Cell/µL) Range**	228	(0–2895)					< 0.0001
< 50	1239	12.8%	1474	22.1%	2713	16.6%	
50–99	1556	16.1%	1302	19.5%	2858	17.5%	
100–199	1577	16.3%	1227	18.4%	2804	17.2%	
200–349	3145	32.6%	1520	22.8%	4665	28.6%	
350–499	909	9.4%	447	6.7%	1356	8.3%	
500 +	1231	12.7%	694	10.4%	1925	11.8%	
**Previous ART exposition**							< 0.0001
ART naive	9274	96.0%	6180	92.7%	15,454	94.7%	
ART non-naive	383	4.0%	484	7.3%	867	5.3%	
**Anti-TB Treatment**							< 0.0001
Complete ATT before ART initiation	252	2.7%	259	4.1%	511	3.3%	
Initiated ATT < 90 days after ART initiation	2064	22.2%	2603	40.8%	4667	29.8%	
Unexposed to ATT initiation	6979	75.1%	3511	55.1%	10,490	67.0%	
**WHO clinical staging of HIV disease**							< 0.0001
Clinical stage I	3168	32.8%	1333	20.0%	4501	27.6%	
Clinical stage II	2478	25.7%	1110	16.7%	3588	22.0%	
Clinical stage III	1517	15.7%	1220	18.3%	2737	16.8%	
Clinical stage IV	2494	25.8%	3001	45.0%	5495	33.7%	
**Kaposi Sarcoma**							< 0.0001
Kaposi Sarcoma	137	1.4%	162	2.4%	299	1.8%	
No Kaposi Sarcoma	9520	98.6%	6502	97.6%	16,022	98.2%	
**ART enrollment year**							< 0.0001
2002	5	0.1%	3	0.0%	8	0.0%	
2003	42	0.4%	35	0.5%	77	0.5%	
2004	36	0.4%	27	0.4%	63	0.4%	
2005	145	1.5%	89	1.3%	234	1.4%	
2006	494	5.1%	291	4.4%	785	4.8%	
2007	776	8.0%	456	6.8%	1232	7.5%	
2008	820	8.5%	462	6.9%	1282	7.9%	
2009	812	8.4%	461	6.9%	1273	7.8%	
2010	846	8.8%	623	9.3%	1469	9.0%	
2011	686	7.1%	519	7.8%	1205	7.4%	
2012	812	8.4%	586	8.8%	1398	8.6%	
2013	632	6.5%	398	6.0%	1030	6.3%	
2014	1054	10.9%	678	10.2%	1732	10.6%	
2015	842	8.7%	573	8.6%	1415	8.7%	
2016	652	6.8%	512	7.7%	1164	7.1%	
2017	428	4.4%	416	6.2%	844	5.2%	
2018	358	3.7%	316	4.7%	674	4.1%	
2019	217	2.2%	219	3.3%	436	2.7%	
**ART therapy initiation**							< 0.0001
ABC + 3TC + EFV	31	0.3%	25	0.4%	56	0.3%	
ABC + 3TC + LPV/RTV	8	0.1%	8	0.1%	16	0.1%	
AZT + 3TC + ABC	181	1.9%	22	0.3%	203	1.2%	
AZT + 3TC + EFV	121	1.3%	297	4.5%	418	2.6%	
AZT + 3TC + LPV/RTV	27	0.3%	25	0.4%	52	0.3%	
AZT + 3TC + NVP	3486	36.1%	2025	30.4%	5511	33.8%	
D4T + 3TC + ABC	490	5.1%	41	0.6%	531	3.3%	
D4T + 3TC + EFV	38	0.4%	628	9.4%	666	4.1%	
D4T + 3TC + LPV/RTV	2	0.0%	1	0.0%	3	0.0%	
D4T + 3TC + NVP	959	9.9%	629	9.4%	1588	9.7%	
TDF + 3TC + DTG	27	0.3%	41	0.6%	68	0.4%	
TDF + 3TC + EFV	3996	41.4%	2771	41.6%	6767	41.5%	
TDF + 3TC + LPV/RTV	291	3.0%	151	2.3%	442	2.7%	

^1^*p* values based on chi-squared test for any difference across groups over both gender status.

*ART* antiretroviral treatment, *ABC* abacavir, *3TC* lamivudine, *EFV* efavirenz, *LPV/RTV* lopinavir/ritonavir, *AZT* zidovudine, *D4T* stavudine; *NVP* nevirapine, *DGT* dolutegravir.

### Patient characteristics by CD4 count, WHO clinical stage, and ATT exposure

Of the total sample, 16.6% (n = 2713) of the patients initiated ART with CD4 counts of less than 50 cells/µL. Over half of these (n = 1474, 54.3%) were men, 39.6% (n = 1071) were aged 25–34 years, 39.6% (n = 1075) presented to CHC to initiate ART as inpatients; 64.7% (n = 1654) started ATT within 90 days of ART initiation, and 66.9% (n = 1814) presented with WHO clinical stage I disease (Table 3).

**Table 3:** Characteristics of HIV patients enrolled at Carmelo Hospital of Chókwè between January 1, 2002 and December 31, 2019 by CD4 Cells count status.

	CD4 cell count per microliter													
	< 50		50–99		100–199		200–349		350–499		500 +		Total	
	N	%	N	%	N	%	N	%	N	%	N	%	N	%
**CD4 + T-cell count (Cell/µL) , median (IQR)**	17	(0–49)	87	(50–99)	146	(100–199)	279	(200–349)	418	(350–499)	829	(500–2895)	192	(0–2895)
**Gender**														
Female	1239	45.7%	1556	54.4%	1577	56.2%	3145	67.4%	909	67.0%	1231	63.9%	9657	59.2%
Male	1474	54.3%	1302	45.6%	1227	43.8%	1520	32.6%	447	33.0%	694	36.1%	6664	40.8%
**Age (years) , median (IQR)**	35	(15–88)	36	(15–93)	35	(15–90)	34	(15–91)	34	(15–79)	34	(15–83)	35	(15–93)
**Age band**														
15–24	212	7.8%	190	6.6%	244	8.7%	560	12.0%	209	15.4%	282	14.6%	1697	10.4%
25–34	1071	39.5%	1051	36.8%	1046	37.3%	1801	38.6%	517	38.1%	704	36.6%	6190	37.9%
35–44	880	32.4%	899	31.5%	791	28.2%	1235	26.5%	372	27.4%	536	27.8%	4713	28.9%
45–54	378	13.9%	485	17.0%	492	17.5%	722	15.5%	170	12.5%	243	12.6%	2490	15.3%
55 +	172	6.3%	233	8.2%	231	8.2%	347	7.4%	88	6.5%	160	8.3%	1231	7.5%
**Point of entry**														
In-patient	1075	39.6%	528	18.5%	377	13.4%	300	6.4%	69	5.1%	82	4.3%	2431	14.9%
Out-patient	1638	60.4%	2330	81.5%	2427	86.6%	4365	93.6%	1287	94.9%	1843	95.7%	13,890	85.1%
**Previous ART exposition**														
ART naive	2533	93.4%	2676	93.6%	2685	95.8%	4525	97.0%	1304	96.2%	1731	89.9%	15,454	94.7%
ART non-naive	180	6.6%	182	6.4%	119	4.2%	140	3.0%	52	3.8%	194	10.1%	867	5.3%
**Previous Anti-TB Treatment**														
Complete ATT before ART initiation	96	3.8%	100	3.7%	116	4.4%	157	3.5%	26	1.9%	16	0.8%	511	3.3%
Initiated ATT < 90 days after ART initiation	1654	64.7%	1066	39.4%	837	31.5%	744	16.5%	193	14.5%	173	9.1%	4667	29.8%
Unexposed to ATT initiation	808	31.6%	1538	56.9%	1706	64.2%	3601	80.0%	1116	83.6%	1721	90.1%	10,490	67.0%
**WHO clinical staging of HIV disease**														
Clinical stage I	186	6.9%	342	12.0%	547	19.5%	1480	31.7%	696	51.3%	1250	64.9%	4501	27.6%
Clinical stage II	233	8.6%	599	21.0%	720	25.7%	1425	30.5%	304	22.4%	307	15.9%	3588	22.0%
Clinical stage III	480	17.7%	625	21.9%	550	19.6%	817	17.5%	128	9.4%	137	7.1%	2737	16.8%
Clinical stage IV	1814	66.9%	1292	45.2%	987	35.2%	943	20.2%	228	16.8%	231	12.0%	5495	33.7%
**Kaposi Sarcoma**														
Kaposi Sarcoma	72	2.7%	66	2.3%	50	1.8%	55	1.2%	23	1.7%	33	1.7%	299	1.8%
No Kaposi Sarcoma	2641	97.3%	2792	97.7%	2754	98.2%	4610	98.8%	1333	98.3%	1892	98.3%	16,022	98.2%
**ART enrollment year**														
2002	3	0.1%	2	0.1%	2	0.1%	1	0.0%	0	0.0%	0	0.0%	8	0.0%
2003	22	0.8%	12	0.4%	24	0.9%	13	0.3%	6	0.4%	0	0.0%	77	0.5%
2004	15	0.6%	10	0.3%	21	0.7%	10	0.2%	5	0.4%	2	0.1%	63	0.4%
2005	66	2.4%	34	1.2%	76	2.7%	48	1.0%	8	0.6%	2	0.1%	234	1.4%
2006	169	6.2%	369	12.9%	131	4.7%	100	2.1%	14	1.0%	2	0.1%	785	4.8%
2007	176	6.5%	722	25.3%	174	6.2%	149	3.2%	7	0.5%	4	0.2%	1232	7.5%
2008	254	9.4%	144	5.0%	277	9.9%	563	12.1%	37	2.7%	7	0.4%	1282	7.9%
2009	229	8.4%	158	5.5%	300	10.7%	531	11.4%	49	3.6%	6	0.3%	1273	7.8%
2010	256	9.4%	189	6.6%	321	11.4%	661	14.2%	34	2.5%	8	0.4%	1469	9.0%
2011	226	8.3%	190	6.6%	229	8.2%	508	10.9%	31	2.3%	21	1.1%	1205	7.4%
2012	262	9.7%	189	6.6%	261	9.3%	547	11.7%	77	5.7%	62	3.2%	1398	8.6%
2013	148	5.5%	109	3.8%	212	7.6%	408	8.7%	74	5.5%	79	4.1%	1030	6.3%
2014	323	11.9%	215	7.5%	272	9.7%	475	10.2%	371	27.4%	76	3.9%	1732	10.6%
2015	228	8.4%	145	5.1%	261	9.3%	327	7.0%	338	24.9%	116	6.0%	1415	8.7%
2016	231	8.5%	150	5.2%	132	4.7%	232	5.0%	158	11.7%	261	13.6%	1164	7.1%
2017	53	2.0%	105	3.7%	51	1.8%	75	1.6%	117	8.6%	443	23.0%	844	5.2%
2018	33	1.2%	84	2.9%	45	1.6%	8	0.2%	26	1.9%	478	24.8%	674	4.1%
2019	19	0.7%	31	1.1%	15	0.5%	9	0.2%	4	0.3%	358	18.6%	436	2.7%
**ART therapy initiation**														
ABC + 3TC + EFV	22	0.8%	10	0.3%	5	0.2%	6	0.1%	3	0.2%	10	0.5%	56	0.3%
ABC + 3TC + LPV/RTV	5	0.2%	4	0.1%	2	0.1%	1	0.0%	0	0.0%	4	0.2%	16	0.1%
AZT + 3TC + ABC	46	1.7%	26	0.9%	34	1.2%	78	1.7%	10	0.7%	9	0.5%	203	1.2%
AZT + 3TC + EFV	91	3.4%	54	1.9%	73	2.6%	106	2.3%	60	4.4%	34	1.8%	418	2.6%
AZT + 3TC + LPV/RTV	18	0.7%	5	0.2%	11	0.4%	13	0.3%	2	0.1%	3	0.2%	52	0.3%
AZT + 3TC + NVP	662	24.4%	1148	40.2%	1164	41.5%	2151	46.1%	241	17.8%	145	7.5%	5511	33.8%
D4T + 3TC + ABC	188	6.9%	104	3.6%	85	3.0%	121	2.6%	21	1.5%	12	0.6%	531	3.3%
D4T + 3TC + EFV	289	10.7%	146	5.1%	117	4.2%	86	1.8%	16	1.2%	12	0.6%	666	4.1%
D4T + 3TC + LPV/RTV	1	0.0%	0	0.0%	1	0.0%	1	0.0%	0	0.0%	0	0.0%	3	0.0%
D4T + 3TC + NVP	397	14.6%	398	13.9%	327	11.7%	421	9.0%	29	2.1%	16	0.8%	1588	9.7%
TDF + 3TC + DTG	1	0.0%	7	0.2%	4	0.1%	1	0.0%	0	0.0%	55	2.9%	68	0.4%
TDF + 3TC + EFV	907	33.4%	801	28.0%	900	32.1%	1587	34.0%	963	71.0%	1609	83.6%	6767	41.5%
TDF + 3TC + LPV/RTV	86	3.2%	155	5.4%	81	2.9%	93	2.0%	11	0.8%	16	0.8%	442	2.7%
**Outcomes**														
LTFU	597	22.0%	676	23.7%	821	29.3%	1330	28.5%	414	30.5%	293	15.2%	4131	25.3%
Retention	593	21.9%	1173	41.0%	1135	40.5%	2299	49.3%	737	54.4%	1342	69.7%	7279	44.6%
Death	1220	45.0%	594	20.8%	451	16.1%	399	8.6%	62	4.6%	50	2.6%	2776	17.0%
Transfer-out	303	11.2%	415	14.5%	397	14.2%	637	13.7%	143	10.5%	240	12.5%	2135	13.1%

*ART* antiretroviral treatment, *ABC* abacavir, *3TC* lamivudine, *EFV* efavirenz, *LPV/RTV* lopinavir/ritonavir, *AZT* zidovudine, *D4T* stavudine; *NVP* nevirapine, *DGT* dolutegravir.

### Cox proportional hazards model for attrition

Among the 2776 PLWHIV who died, 1448 (52.2%) were men, 982 (35.4%) were aged 25–34 years, 1578 (56.65%) started ART as inpatients, 1220 (43.9%) had CD4 counts of less than 50 cells/µL, 2072 (97.4%) were on ATT within 90 days of ART initiation, 2627 (84.6%) were ART-naïve, 133 (4.8%) had Kaposi’s sarcoma, 2417 (87.1%) presented with WHO stage IV disease, and 638 (23.0%) initiated ART on D4T (stavudine 30 mg) + 3TC (lamivudine 150 mg) + NVP (nevirapine 200 mg) (Table 1).

Overall, the 16,321 adults on ART contributed a total of 72,987 person-years to the study data. The overall attrition rate was 9.46 per 100 person-years. According to the multivariable Cox regression model, women were at lower risk of attrition than men (hazard ratio [HR] 0.930, 95% confidence interval [CI] 0.869–0.996; *p* = 0.039). Patients aged 35–44 years old were at lower risk of attrition compared to those aged 55 years or older (HR 0.83, 95% CI 0.74–0.94, *p* = 0.002).

Those who initiated ART as inpatients carried a three-fold higher risk of attrition compared to outpatients (HR 3.18, 95% CI 2.89–3.50, *p* < 0.001). Receiving ATT within 90 days of ART initiation was associated with a six-fold higher risk of attrition compared to those who were not on ATT (HR 6.53, 95% CI 5.72–7.45, *p* < 0.001). Compared to patients with WHO clinical stage IV disease, every other stage was associated with three times higher risk of attrition (stage I: HR 3.52, 95% CI 2.99–4.13, *p* < 0.001; stage II: HR 3.46, 95% CI 2.96–4.05, *p* < 0.001; stage III: HR 3.75; 95% CI 3.21–4.37, *p* < 0.001). Having Kaposi’s sarcoma was also associated with a higher risk of attrition (HR 1.99, 95% CI 1.65–2.39, *p* < 0.001).

Compared to a CD4 count of more than 500 cells/µL, a CD4 count of less than 50 cells/µL was associated with a higher risk of attrition (HR 1.91, 95% CI 1.63–2.24, *p* < 0.001), while a CD4 count of 350–499 cells/µL was associated with a lower risk (HR 0.79, 95% CI 0.65–0.97, *p* < 0.023) (Table 4).

**Table 4:** Cox proportional hazards model for attrition among PLWH on ART at Carmelo Hospital of Chókwè (2002–2019).

	Total		Attrition		Attrition rate/100 py	Attrition			
	N	%	N	%	(overall = 9.46)	Hazard Ratio	Lower CI	Upper CI	*p* value
**Gender**									
Male	6664	40.8	3471	50.3	7.02	–	–	–	
Female	9657	59.2	3436	49.7	14.43	0.93	0.869	0.996	0.039
**Age (years) , median (IQR)**									
**Age band**									
55 +	1231	7.5	543	7.9	10.49	–	–	–	
15–24	1697	10.4	794	11.5	12.24	1.077	0.935	1.241	0.306
25–34	6190	37.9	2737	39.6	10.34	0.919	0.82	1.031	0.149
35–44	4713	28.9	1871	27.1	8.47	0.832	0.74	0.936	0.002
45–54	2490	15.3	962	13.9	7.53	0.922	0.812	1.046	0.207
**Point of entry**									
Out-patient	13,890	85.1	4983	72.1	7.37	–	–	–	
In-patient	2431	14.9	1924	27.9	35.60	3.18	2.894	3.495	< 0.0001
**CD4 + T-cell count (Cell/µL) , median (IQR)**									
**CD4 + T-cell count (Cell/µL) Range**									
500 +	1925	11.8	343	5.0	8.11	–	–	–	
< 50	2713	16.6	1817	26.3	22.28	1.91	1.626	2.242	< 0.0001
50–99	2858	17.5	1270	18.4	8.20	1.182	1.006	1.388	0.042
100–199	2804	17.2	1272	18.4	9.27	1.171	0.996	1.378	0.057
200–349	4665	28.6	1729	25.0	6.69	1.024	0.874	1.201	0.767
350–499	1356	8.3	476	6.9	8.56	0.794	0.65	0.969	0.023
**Previous ART exposition**									
ART non-naive	867	5.3	437	6.3	21.16	–	–	–	
ART naive	15,454	94.7	6470	93.7	9.12	0.91	0.788	1.051	0.197
**Previous Anti-TB Treatment**									
Unexposed to ATT initiation	10,490	67.0	2938	47.0	5.26	–	–	–	
Complete ATT before ART initiation	511	3.3	206	3.3	7.62	1.476	1.215	1.794	< 0.0001
Initiated ATT < 90 days after ART initiation	4667	29.8	3112	49.7	24.13	6.528	5.722	7.448	< 0.0001
**WHO clinical staging of HIV disease**									
Clinical stage IV	5495	33.7	3642	52.7	22.03	–	–	–	
Clinical stage I	4501	27.6	1141	16.5	5.99	3.516	2.994	4.128	< 0.0001
Clinical stage II	3588	22.0	1089	15.8	4.95	3.464	2.964	4.048	< 0.0001
Clinical stage III	2737	16.8	1035	15.0	6.72	3.746	3.21	4.372	< 0.0001
**Kaposi Sarcoma**									
No Kaposi Sarcoma	16,022	98.2	6694	96.9	9.29	–	–	–	
Kaposi Sarcoma	299	1.8	213	3.1	22.44	1.987	1.652	2.39	< 0.0001
**ART enrollment year**									
2019	436	2.7	53	0.8	20.85	–	–	–	
2002	8	0.0	4	0.1	6.89	0.14	0.054	0.358	< 0.0001
2003	77	0.5	24	0.3	2.74	0.31	0.182	0.526	< 0.0001
2004	63	0.4	27	0.4	4.75	0.415	0.247	0.697	0.001
2005	234	1.4	105	1.5	5.56	0.318	0.216	0.467	< 0.0001
2006	785	4.8	386	5.6	6.93	0.347	0.246	0.491	< 0.0001
2007	1232	7.5	643	9.3	7.89	0.319	0.228	0.448	< 0.0001
2008	1282	7.9	570	8.3	6.65	0.293	0.209	0.411	< 0.0001
2009	1273	7.8	564	8.2	7.32	0.295	0.21	0.414	< 0.0001
2010	1469	9.0	710	10.3	9.53	0.383	0.274	0.534	< 0.0001
2011	1205	7.4	596	8.6	11.03	0.457	0.329	0.635	< 0.0001
2012	1398	8.6	649	9.4	10.57	0.376	0.27	0.522	< 0.0001
2013	1030	6.3	443	6.4	10.42	0.305	0.218	0.427	< 0.0001
2014	1732	10.6	691	10.0	11.03	0.493	0.36	0.676	< 0.0001
2015	1415	8.7	538	7.8	12.24	0.525	0.382	0.72	< 0.0001
2016	1164	7.1	411	6.0	13.92	0.559	0.407	0.769	< 0.0001
2017	844	5.2	305	4.4	18.92	0.761	0.553	1.046	0.092
2018	674	4.1	188	2.7	21.54	0.98	0.706	1.36	0.904
**ART therapy initiation**									
TDF + 3TC + LPV/RTV	442	2.7	145	2.1	3.50	–	–	–	
ABC + 3TC + EFV	56	0.3	30	0.4	31.57	3.978	2.589	6.111	< 0.0001
ABC + 3TC + LPV/RTV	16	0.1	11	0.2	10.54	2.189	1.05	4.561	0.037
AZT + 3TC + ABC	203	1.2	109	1.6	12.40	2.984	2.152	4.139	< 0.0001
AZT + 3TC + EFV	418	2.6	194	2.8	12.79	2.371	1.757	3.201	< 0.0001
AZT + 3TC + LPV/RTV	52	0.3	19	0.3	4.07	0.695	0.336	1.438	0.327
AZT + 3TC + NVP	5511	33.8	1960	28.4	5.11	1.851	1.479	2.317	< 0.0001
D4T + 3TC + ABC	531	3.3	406	5.9	34.91	6.497	5.055	8.35	< 0.0001
D4T + 3TC + EFV	666	4.1	556	8.0	70.37	7.842	6.127	10.036	< 0.0001
D4T + 3TC + LPV/RTV	3	0.0	2	0.0	6.78	0.778	0.107	5.634	0.803
D4T + 3TC + NVP	1588	9.7	1169	16.9	35.62	6.934	5.502	8.738	< 0.0001
TDF + 3TC + DTG	68	0.4	1	0.0	3.92	0.477	0.113	2.004	0.312
TDF + 3TC + EFV	6767	41.5	2305	33.4	10.41	1.895	1.483	2.421	< 0.0001

^1^*p*-values based on Cox regression proportional hazard risk.

*ART* antiretroviral treatment, *ABC* abacavir, *3TC* lamivudine, *EFV* efavirenz, *LPV/RTV* lopinavir/ritonavir, *AZT* zidovudine, *D4T* stavudine; *NVP* nevirapine, *DGT* dolutegravir.

Women, patients who were ART-naïve, those with CD4 count of more than 200 cells/µL at ART enrollment year, and certain drug combinations (ABC/AZT/D4T + 3TC + LPV/RTV,) were not associated with higher mortality (Table 4)*.*

### Survival estimates

**Figure 3:**
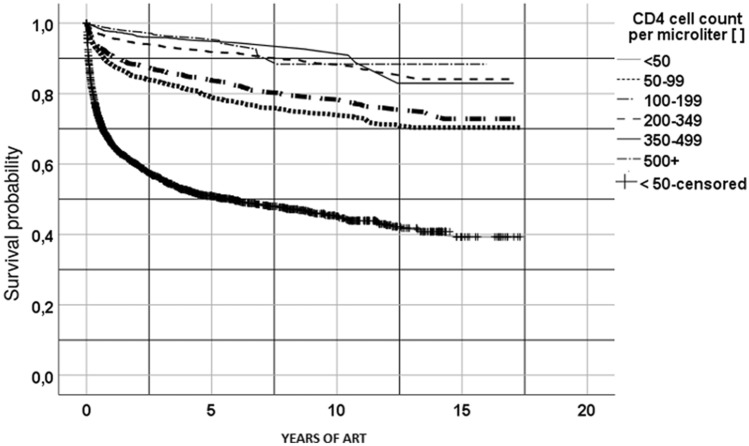
Kaplan–Meier plot for PLHIV on ART at Carmelo Hospital of Chókwè (2002–2019), by CD4 cell count per µL.

Kaplan–Meier estimates for the probability of 18-year survival by CD4 cell count on ART initiation are presented in Fig. 3.

PLWHIV who had a CD4 count of more than 500 cells/µL had the highest probability of approximately 90% of survival at 18 years following ART initiation, while those with a CD4 count of less than 50 cells/µL had a survival probability of approximately 40% at 18 years in comparison.

## Discussion

In our cohort, patients with CD4 counts of less than 50 cells/µL had almost double the attrition risk compared to those with CD4 counts of more than 500 cells/µL. These findings are a little higher than observed in other Mozambican literature^21^. An in-depth analysis of our cohort showed that the frequency of attrition in the patients with low CD4 cell counts was 26.3%; of these, 43.9% died, and 14.5% were lost to follow-up. Other studies have reported high mortality and LTFU in severely immunosuppressed patients^22,23^, highlighting LTFU as a fundamental problem for the health care in PLHIV. Risk factors for ART interruption are still poorly understood in many settings, including Mozambique.

The survival analysis shows that in our cohort, patients with CD4 counts under 50 cells/µL had an 18-year survival rate of less than 50%. These data are consistent with other reports^24^. The baseline CD4 cell count is the dominant prognostic factor for survival in PLHIV starting ART^25^. Our study showed that patients who started treatment at an advanced disease stage are at a higher risk of mortality than those who start treatment with higher CD4 cell counts, even if they survive the first 5 years of therapy. These findings are in keeping with studies from high-income settings^25^.

On analyzing the first 2 years of follow-up, we observed a considerable drop in the survival curve among people with CD4 counts of less than 50 cells/µL, from 100 to 60%; by 5 years, survival stood at just 50%. This finding is strongly related to the fact that severe immunosuppression in this population is not restricted to HIV infection alone; rather, multiple factors are at play, including hunger, adult malnutrition^26^, and poor nutritional supplementation^27^. These people are considered “immunological non-responders,” that is, HIV-infected individuals who fail to achieve normalization of CD4^+^ T-cell counts despite persistent virological suppression, which leads to AIDS and non-AIDS comorbidities^28,29^. This population continues to have a substantially increased mortality ratio even after 3 years of treatment. Thus, clinical factors such as immunological non-response, AIDS and non-AIDS-related multiple events predict a substantially increased risk of death after 3 years of ART treatment^30,31^.

Recovery of CD4 lymphocytes occurs in the first 2 years after starting ART and is associated with age and the pre-existing degree of HIV-1-related immunodeficiency, so long-term exposure to HIV-1 infection damages the immune system in ways that are difficult to correct due to exhaustion^32–34^.

Better understanding of patterns and risk factors that cause-specific mortality in the ART era can inform the development of appropriate care for HIV-infected individuals and guidelines for risk factor management^35^. This may include screening programs and intensive adherence counseling^34^.

We observed that patients who received ATT within the first 90 days of their ART initiation had a six-fold higher risk of attrition than those who were unexposed to ATT. These results are a little higher than those observed in other Mozambican studies^36^. In our cohort, the attrition rate on active TB patients was 24.13 per 100 person-years, and the frequency of attrition was 49.7%. The overwhelming majority of patients receiving ATT within 90 days (97.4%) died, while 25.3% of those LTFU had a fatal outcome. These results show high mortality but low LTFU, so having TB is a favorable predictor against LTFU.

In our sample, men were underrepresented (39%), as published elsewhere^37^. There were few men who went to health facilities to test for HIV and to initiate and adhere to ART^38,39^. We fully acknowledge that more women than men take an active role in accessing HIV services, and studies show that their adherence to ART is also higher^40^. This tendency may be rooted in different dimensions of gender norms in rural areas^41^.

Our cohort shows a higher ART initiation rate, of around 37.9%, and a higher death rate among 25–34-year olds (35.4%) compared to an Ethiopian University Teaching Hospital study, which reported an ART initiation rate of 27.9% in the same age group^24^. Just over 20% of men initiated ART as inpatients, compared to about 11% of women. Similarly, 22.1% of men started ART with CD4 counts of less than 50 cells/µL, compared to 12.8% of women, in keeping with reports elsewhere^25^.

With this advanced immune suppression, then, a high proportion of men started ART with advanced AIDS and AIDS Clinical Stage IV (45.0% in men vs. 25.8% in women). Immune reconstitution syndrome was closely associated with tuberculosis, confirming previous findings from our cohort and other resource-limited settings^42,43^. This syndrome was more frequent in men (40.8% vs 22.2%), and over the course of 18 years it was observed that the male population was always on a smaller scale.

Women comprised the majority of the population at 60% compared to men, but half (49%) were classified in the attrition group. Being a woman was not a statistically significant favorable predictor in our cohort.

Among the patient group with CD4 counts of less than 50 cells/µL, over half were men, 39.55% were aged 25–34 years, and 65% had started ATT within 90 days of initiating ART. These findings are consistent with data from other studies^44,45^, which reveal a greater predominance of men among patients with late HIV diagnosis as well as late-onset ART. The care, ART initiation and treatment monitoring are in accordance with the WHO and national ART guidelines, which were updated successively in 2002^46^, 2009^47^, 2013^48^, 2016^49^, 2017^50^ and 2019^51^. The number of participants changes over time according to these updates (Supplementary Table). From 2002 to 2008 (Era CD4 < 200 cell count^46^), there was a progressive increase in the number of patients starting ART and a stabilization in the number of new ART initiations in the latest years of the regimen. Both in Era CD4 < 250 cell count (years 2009–2012^47^) and Era CD4 < 350 cell count (years 2013–2015)^48^), there was a considerable increase in recruitment at 1 year after the implementation of the new ART eligibility criteria, which subsequently decreased, in part due to the expansion of ART services in several other new peripheral health facilities. In the CD4 < 500 era (years 2017–2018^50^) there was no increase but a continued decrease in recruitment, a reflection of the efforts of the Ministry of Health and cooperating partners (the Global Fund, and PEPFAR) to expand the health network that allows for the distribution of patients to other peripheral facilities.

In 2006/2007^46^, the number of new patients who started ART doubled compared to the years 2004/2005 (Supplementary Table S1). This is partly due to community awareness campaigns on ART treatment, as well as the influx of patients into inpatient services. At that time, many patients were diagnosed at the advanced stage of the AIDS disease, consequently with severe immunosuppression.

The years 2014/2015^48^ were the first following implementation of other eligibility criteria that defined co-infection with TB, Hepatitis B, cancer, and HTLV as criteria for starting ART, regardless of WHO stage or CD4 cell count (Supplementary Table S1). Thus, these data contributed to a substantial increase in patients with CD4 counts of 350–399 cells/µL.

The now outdated D4T-based regime was used as a first-line treatment from 2002 to 2018^46–50^, and as an alternative line in patients with Hgb anemia of less than 8 g/dL at the time of screening. Another outdated initial regime, based on AZT, was used from 2009 to 2018^47–50^. The updated TDF-based regime has been used since 2013^48^, and the current DTG-based regime since 2019^51^, a period characterized by the definitive discontinuation of INNRT (NVP/EFV) drugs.

The strengths of this study reside in the large sample size and the long study period. Limitations include those of many retrospective studies in which data collection is often inadequate and incomplete, without baseline viral load and data to monitor therapeutic failure. This may have an influence on the reported results, mainly for death and LTFU.

## Conclusion

The results of this cohort study indicate that attrition after ART initiation is high. Nearly 40% of patients die or are LTFU. Low CD4 cell counts, starting ART as an inpatient, WHO clinical stage IV, and being a man were strongly associated with attrition. Although the mortality of patients diagnosed with tuberculosis after ART initiation was extremely high, patients who started anti-tuberculosis treatment before ART had similar attrition as patients without tuberculosis. The results of this study indicate that interventions aimed at initiating patients sooner after HIV seroconversion, improving the diagnosis and treatment of TB and other comorbidities (like Kaposi’s sarcoma), and giving extra support to patient groups at higher risk of attrition could potentially reduce the mortality and LTFU in HIV programs in Mozambique.

## Supplementary Information

Supplementary Information.

## Data Availability

The datasets analyzed during the current study are not publicly available but are available from the corresponding author on reasonable request.

## Abbreviations

3TC: Lamivudine
ABC: Abacavir
ART: Antiretroviral therapy
ATT: Anti tuberculosis treatment
AZT: Zidovudine
BMI: Body mass index
CHC: Carmelo Hospital Chókwè
D4T: Stavudine
DGT: Dolutegravir
EFV: Efavirenz
HR: Hazard ratio
LPV/RTV: Lopinavir/ritonavir
LTFU: Loss to follow up
NVP: Nevirapine
PEPFAR: President’s emergency plan for AIDS relief
PLWHIV: People living with HIV
Person-years: Person-years of observed therapy
UNAIDS: United Nations Programme on HIV/AIDS
WHO: World Health Organization
