# Supplementary material for: Predictors of attrition among adults in a rural HIV clinic in southern Mozambique: 18-year retrospective study
Source: Sci Rep. 2021 Sep 9;11:17897. doi: 10.1038/s41598-021-97466-2 (PMC8429703; doi:10.1038/s41598-021-97466-2)
Supplement: Supplementary file 1 — Supplementary Information. [file 41598_2021_97466_MOESM1_ESM.docx]

**Supplementary Table : Mozambican antiretroviral therapy (ART) eligibility guidelines for adults (2002-2019)**

| **Era** | **Eligibility criteria** | **1^st^ ART Lines** | **2^nd^ ART Lines** |
| --- | --- | --- | --- |
| 2002 [47] | -WHO stage 4 regardless of CD4 cell count  -CD4 < 200 Cell/µL  -CD4 between 200-350 Cell/µL and WHO stage 3  World Health Organization. (2002). *Scaling up antiretroviral therapy in resource-limited settings: guidelines for a public health approach: executive summary* (No. WHO/HIV/2002.01). World Health Organization. | -D4T+3TC+NVP/EFV | -ABC+3TC+LPV/RTV |
| 2009 [48] | -WHO stage 4 regardless of CD4 cell count  -CD4 < 250 cell count regardless of WHO stage  -Pregnancy with CD4 < 350 Cell/µL  *GUIA DE TRATAMENTO ANTIRETROVIRAL E INFECÇÕES OPORTUNISTAS NO ADULTO, ADOLESCENTE E GRÁVIDA. 2009.* [*https://www.who.int/hiv/pub/guidelines/mozambique_art.pdf*](https://www.who.int/hiv/pub/guidelines/mozambique_art.pdf) | -AZT + 3TC + NVP  -ALTERNATIVES FOR THE 1st LINE:  -If anemia with Hgb ≤ 8g / dl: d4T + 3TC + NVP  -If it is intolerance to NVP Grade 1 or 2: monitor the evolution of the condition. If it persists or worsens after 2 weeks, switch NVP to EFV  -If intolerance to NVP Grade 3 or 4: AZT + 3TC + ABC  - If anemia with Hgb ≤ 8g / dl AND peripheral neuropathy: TDF + 3TC + NVP | -ABC+3TC+LPV/RTV  -D4T+3TC+LPV/RTV |
| 2013 [49] | -WHO stage 3 and 4 regardless of CD4 cell count  -CD4 < 350 cell count regardless of WHO stage  -WHO stage 4 regardless of CD4 cell count  -Pregnancy and breastfeeding regardless of WHO stage and CD4 cell count  -Co-infection with TB, Hepatitis B, cancer, HTLV regardless of WHO stage and CD4 cell count  -Serodiscordant couples: prioritizing seropositive partners of seronegative pregnant women  *Orientação novas normas TARV adultos 23-04-2013.* [*https://comitetarvmisau.co.mz/docs/orientacoes_nacionais/Orientacao%20novas%20normas%20TARV%20pediatrico%2029-05-2013.pdf*](https://comitetarvmisau.co.mz/docs/orientacoes_nacionais/Orientacao%20novas%20normas%20TARV%20pediatrico%2029-05-2013.pdf) | -TDF+3TC+EFV  -If it is intolerance to TDF Grade 1 or 2: Switch to AZT+3TC+NVP or ABC+3TC+NVP or AZT+3TC+EFV or ABC+3TC+EFV or D4T+3TC+EFV or D4T+3TC+NVP | -ABC+3TC+LPV/RTV  -AZT+3TC+LPV/RTV  -D4T+3TC+LPV/RTV |
| 2016 [50] | -WHO stage 3 and 4 regardless of CD4 cell count  -CD4 < 500 cell count regardless of WHO stage  -WHO stage 4 regardless of CD4 cell count  -Pregnancy and breastfeeding regardless of WHO stage and CD4 cell count  -Co-infection with TB, Hepatitis B, cancer, HTLV regardless of WHO stage and CD4 cell count  -Serodiscordant couples: prioritizing seropositive partners of seronegative pregnant women  *Norma TARV adultos Março 2016* [*https://comitetarvmisau.co.mz/docs/orientacoes_nacionais/NORMA_TARV_ADULTOS_MARCO_2016.pdf*](https://comitetarvmisau.co.mz/docs/orientacoes_nacionais/NORMA_TARV_ADULTOS_MARCO_2016.pdf) |  |  |
| 2017 [51] | -Test and Treat  *Circular Testar e Tratar fase 2 Fevereiro 2017* [*https://comitetarvmisau.co.mz/docs/orientacoes_nacionais/circular_testar_e_Iniciar_fase%202.pdf*](https://comitetarvmisau.co.mz/docs/orientacoes_nacionais/circular_testar_e_Iniciar_fase%202.pdf) |  |  |
| 2019 [52] | -Test and Treat  ART should be initiated in all adults living with HIV, regardless of WHO clinical stage and at any CD4 cell count  *Circular Normas Clínicas 08.03.19* [*https://comitetarvmisau.co.mz/docs/orientacoes_nacionais/Circular_Normas_Cl%C3%ADnicas_Actualizadas_29_11_19.pdf*](https://comitetarvmisau.co.mz/docs/orientacoes_nacionais/Circular_Normas_Cl%C3%ADnicas_Actualizadas_29_11_19.pdf) | -TDF+3TC+DTG | -ABC+3TC+LPV/RTV |

3TC: lamivudine, ABC: abacavir, AZT: zidovudine, D4T: stavudine; DGT: dolutegravir, EFV: efavirenz, LPV/RTV: lopinavir/ritonavir, ,WHO: World Health Organization,
